# Successful Management of Subarachnoid Hemorrhage Complicated by Takotsubo Cardiomyopathy Using Distal Transradial Access (dTRA) Coiling and Integrated Pharmacotherapy Under Intra-aortic Balloon Pumping (IABP) Support: A Stroke-Heart Syndrome Case

**DOI:** 10.7759/cureus.86068

**Published:** 2025-06-15

**Authors:** Yu Okuma, Kazumoto Suzuki, Kentaro Shimoda, Akane Tanda, Goro Kido, Yukihide Kagawa

**Affiliations:** 1 Department of Neurological Surgery, Sonoda Daiichi Hospital, Adachi, JPN

**Keywords:** angiotensin receptor-neprilysin inhibitors, beta-blockers, clazosentan, distal trans-radial access, intra-aortic balloon pumping, mineralocorticoid receptor antagonists, perampanel, sah- subarachnoid hemorrhage, takotsubo cardiomyopathy (tc)

## Abstract

Takotsubo cardiomyopathy (TCM) complicating subarachnoid hemorrhage (SAH) presents unique management challenges due to the risk of cardiogenic shock and cerebral ischemia. We report the case of a 55-year-old woman with SAH and severe TCM treated successfully through multidisciplinary care involving intra-aortic balloon pumping (IABP), endovascular aneurysm coiling via distal transradial access (dTRA), and a structured pharmacologic strategy, following hemodynamic stabilization, that included angiotensin receptor-neprilysin inhibitors (ARNIs), beta-blockers (BBs), mineralocorticoid receptor antagonists (MRAs), clazosentan, and perampanel. This case illustrates the feasibility of managing complex neurocardiac comorbidity using integrative, anticipatory strategies in a high care unit (HCU) managed directly by neurosurgeons, outside a formal intensive care unit (ICU).

## Introduction

Takotsubo cardiomyopathy (TCM), as a transient left ventricular dysfunction, often triggered by acute stress, can occasionally complicate subarachnoid hemorrhage (SAH), resulting in cardiogenic shock [1]. In such settings, surgical treatment of ruptured aneurysms is often complicated, especially when intra-aortic balloon pumping (IABP) is required [2]. Moreover, vasospasm therapy with agents such as clazosentan (Idorsia Pharmaceuticals Ltd., Allschwil, Switzerland), as an endothelin receptor antagonist, is frequently withheld due to concerns about fluid retention in patients with impaired cardiac function [3]. Distal transradial access (dTRA) offers a practical alternative to the conventional femoral approach, minimizing access-site complications and avoiding interference with devices such as IABP [4]. We report this case to highlight the successful integration of multidisciplinary therapies, including IABP, distal transradial neurointervention, and careful pharmacologic management, treating the rare and challenging combination of SAH complicated by TCM.

## Case presentation

**Figure 1:**
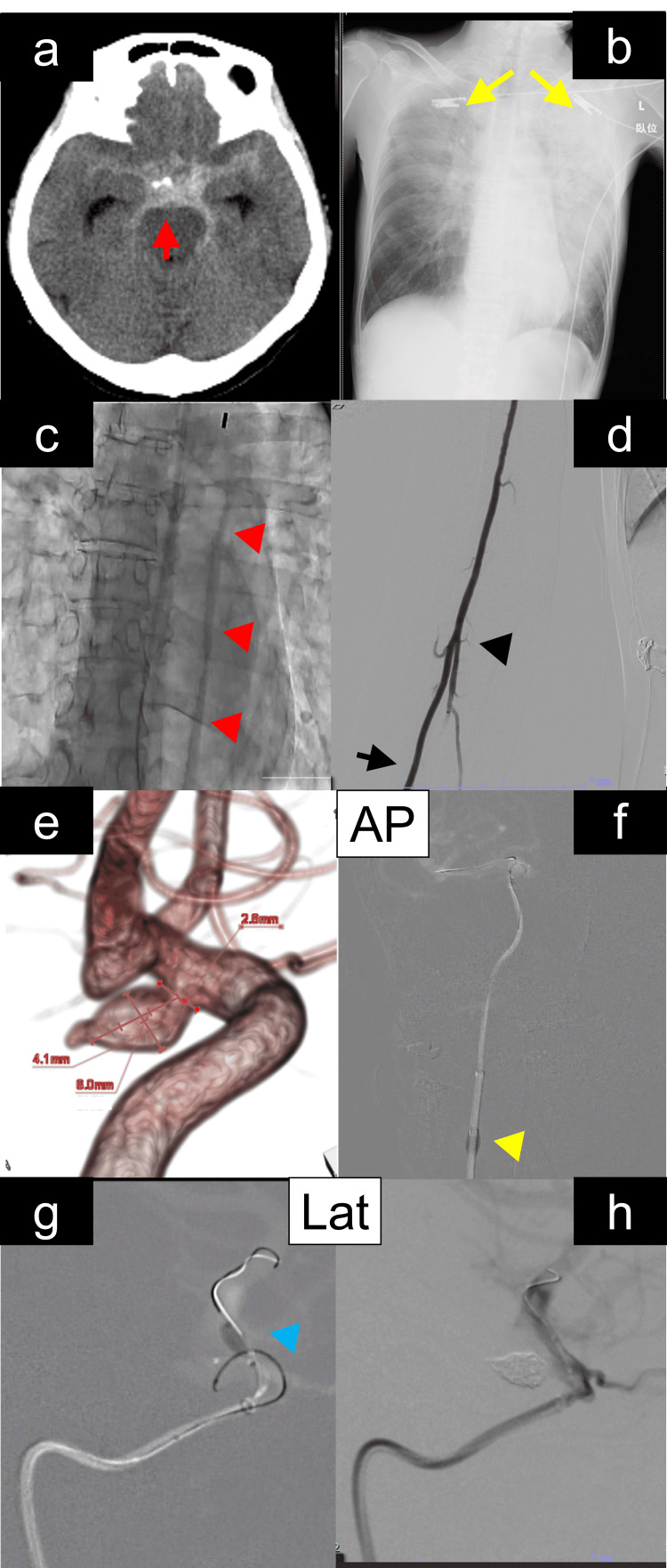
Radiological and procedural findings in a patient with subarachnoid hemorrhage, Takotsubo cardiomyopathy, and aneurysmal embolization under intra-aortic balloon pump (IABP) support a) Initial non-contrast head computed tomography revealed a subarachnoid hemorrhage predominantly in the basal cisterns (red arrow). b) Chest radiography showed signs consistent with Takotsubo cardiomyopathy and pulmonary edema (yellow arrow). c) IABP was placed via the right femoral artery, with the balloon positioned in the descending thoracic aorta (red arrowhead). d) Neuroendovascular intervention was performed via a distal radial artery approach while reviewing the vascular anatomy of the radial (black arrow), brachial artery, and the cubital portion (black arrowhead). e) Cerebral angiography demonstrated a right internal carotid-posterior communicating artery aneurysm with a bleb, measuring 8.0 mm in maximum diameter (excluding the bleb: 6.0 × 4.0 × 3.0 mm; neck: 2.8 mm). f) Proximal flow control was achieved using a balloon guiding catheter (yellow arrowhead). g) Distal flow control was established using a micro-balloon catheter (blue arrowhead). h) Complete flow arrest within the aneurysmal sac was confirmed with no contrast opacification observed. AP: anteroposterior, IABP: intra-aortic balloon pumping, Lat: lateral

A 55-year-old woman with a history of catheter ablation for paroxysmal supraventricular tachycardia three months prior presented with two syncopal episodes at home without focal deficits. On arrival, she was in a pre-shock state with hypotension (blood pressure (BP) ≤ 90 mmHg), tachycardia (≥110 bpm), and oxygen saturation (SpO₂) in the low 90%. Non-contrast computed tomography (CT) revealed SAH in the basal cisterns (Figure 1a), while chest X-ray and CT demonstrated pulmonary edema and pleural effusion (Figure 1b). Contrast-enhanced head CT angiography identified a ruptured right internal carotid-posterior communicating artery (IC-PC) aneurysm. She was diagnosed with SAH due to a ruptured IC-PC aneurysm (World Federation of Neurosurgical Societies (WFNS) grade III, Hunt and Kosnik grade III).

Following emergent intubation and initiation of mechanical ventilation, intravenous norepinephrine was administered. Despite this, her condition deteriorated to shock vitals (BP ≤ 80 mmHg, SpO₂ in the low 80%, heart rate ≥ 130 bpm). Transthoracic echocardiography revealed global left ventricular hypokinesis with a preserved posterior wall, consistent with TCM, and a visual ejection fraction of 25%. Coronary angiography ruled out significant coronary artery disease. Based on these findings, a diagnosis of TCM with pulmonary edema was made. An IABP was inserted via the right femoral artery into the descending thoracic aorta and initiated at a 1:1 assist ratio (Figure 1c). A Swan-Ganz catheter was inserted, showing a cardiac output of 3.5 L/minute, mean pulmonary artery pressure of 28 mmHg, pulmonary capillary wedge pressure (PCWP) of 19 mmHg, and central venous pressure (CVP) of 12 mmHg. Norepinephrine was administered at 0.1-0.2 μg/kg/minute to maintain mean arterial pressure (MAP) >80 mmHg. No inotropes were used aside from mechanical support with IABP.

The next morning, due to operator experience and to preserve the femoral route for IABP, coil embolization was performed using right distal radial artery access (Figure 1d) [4]. An 8Fr balloon guiding catheter was inserted directly via the long dilator kit, followed by intravenous administration of 3,000 units of heparin to maintain an activated clotting time (ACT) of around 200 seconds. After positioning the guiding catheter in the cervical internal carotid artery (ICA), digital subtraction angiography confirmed an IC-PC aneurysm with a bleb (Figure 1e). A 6Fr distal access catheter was advanced to the C4 segment of the right ICA, and a micro balloon catheter was positioned distal to the aneurysm using the microwire. A microcatheter was advanced into the aneurysm with a micro guidewire. Throughout the procedure, IABP support was continued. Despite stable hemodynamics, the aneurysm demonstrated strong pulsatility. Therefore, the microcatheter was carefully positioned within the aneurysm with proximal control using the balloon guiding catheter (Figure 1f) and distal control using the micro balloon catheter (Figure 1g). This dual-balloon technique minimized pulsatility effects exacerbated by TCM. Three platinum coils were inserted; coil prolapse and a tendency for finishing toward the bleb were noted. The balloon position and inflation were repeatedly adjusted to optimize embolization, and a total of 29 cm of coils were deployed. Hemodynamics remained stable intraoperatively (MAP 85-95 mmHg, cardiac output 3.8 L/minute) under IABP support. Post-embolization angiography confirmed complete occlusion of the dome and bleb (Figure 1h). 

**Figure 2:**
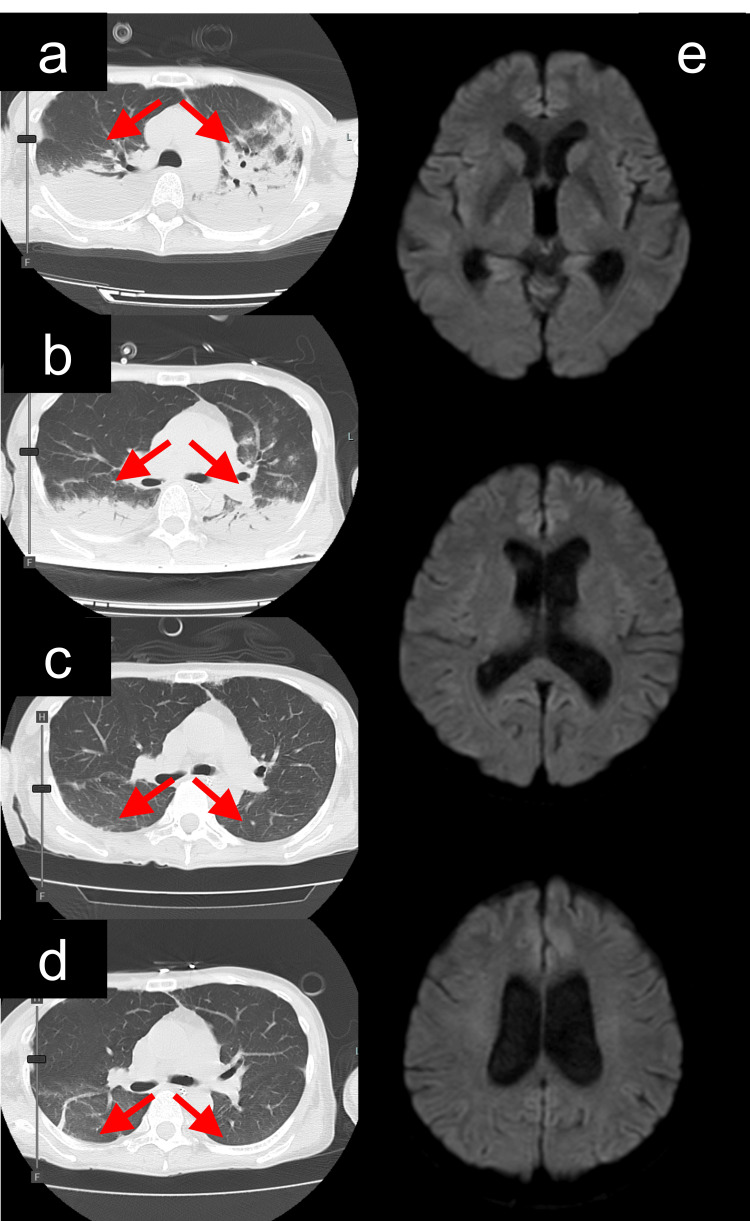
Serial studies demonstrating resolution of pulmonary edema (red arrow) and prevention of vasospasm despite following clazosentan administration a) Chest computed tomography (CT) performed immediately after coil embolization demonstrated still pulmonary edema (red arrow). b) Chest CT obtained on postoperative day (POD) 4 showed a reduction in pulmonary edema (red arrow). c) Chest CT on POD 7, corresponding to day 4 of clazosentan administration, demonstrated resolution of pulmonary edema (red arrow). d) Chest CT on POD 11, also corresponding to day 8 of clazosentan administration, confirmed sustained resolution of pulmonary edema (red arrow). e) Brain magnetic resonance imaging (MRI) performed on POD 11 revealed no evidence of delayed cerebral ischemia (DCI).

She immediately managed after SAH with argatroban anticoagulation during IABP support to manage potential thromboembolic complications. To minimize hemorrhagic risk in the acute post-hemorrhagic period, activated partial thromboplastin time (aPTT) was strictly monitored, with target prolongation maintained at approximately 1.5 times baseline and never exceeding twice the normal value. No hemorrhagic complications were observed throughout the anticoagulation period. Immediately after coil embolization, perampanel (2 mg/day) was initiated prophylactically for seizure prevention via AMPA receptor antagonism during the cerebral vasospasm phase. In addition to standard seizure prophylaxis, this choice was guided by recent evidence suggesting that perampanel may have protective effects against cerebral vasospasm and delayed cerebral ischemia (DCI), although this remains experimental. Norepinephrine was discontinued the same day. IABP was removed on postoperative day (POD) 3, and the patient was extubated on POD 4. Clazosentan administration began on post-extubation day 1 (POD 4) after achieving hemodynamic stabilization, minimizing the risk of fluid retention. Heart failure medications, including angiotensin receptor-neprilysin inhibitors (ARNIs), beta-blockers (BBs), and mineralocorticoid receptor antagonists (MRAs), were introduced. Follow-up angiography on POD 10 and magnetic resonance imaging with diffusion weighted imaging (DWI) and fluid attenuated inversion recovery (FLAIR) sequences and angiography on POD 11 showed no evidence of DCI (Figures 2a-2e) and sustained aneurysm occlusion. The patient was transferred for rehabilitation with a Modified Rankin Scale (mRS) 1 on POD 18 and returned to work one month post-onset with full neurological and cardiac recovery. The clinical timeline of events is presented in Table 1.

**Table 1:** Clinical timeline of events ACT: activated clotting time, ARNI: angiotensin receptor-neprilysin inhibitor, BB: beta-blocker, DSA: digital subtraction angiography, DWI: diffusion-weighted imaging, FLAIR: fluid-attenuated inversion recovery, IABP: intra-aortic balloon pumping, MRA (day 4): magnetic resonance angiography, MRA (day 11): mineralocorticoid receptor antagonist, MRS: Modified Rankin Scale, TCM: Takotsubo cardiomyopathy

Day	Event Description
Day 0	Presentation with syncope and shock; intubation and mechanical ventilation; initial norepinephrine administration; TCM diagnosis; IABP insertion via femoral artery
Day 1	Endovascular coil embolization via right distal radial artery access; intraoperative heparinization (ACT ~200 seconds); postoperative continuous argatroban; perampanel initiated for seizure/vasospasm prevention
Day 3	IABP removed; argatroban discontinued; prasugrel 3.75 mg started for antiplatelet therapy
Day 4	Extubation; start of clazosentan; initiation of heart failure medications (ARNI, BB, MRA)
Day 10	Follow-up DSA (angiography) - no recanalization or vasospasm
Day 11	Follow-up brain MRI (DWI/FLAIR/MRA) - no DCI; pulmonary edema resolved
Day 18	Transferred to rehabilitation facility (mRS 1)
Day 30	Outpatient follow-up; returned to work (mRS 0); switched from argatroban to oral prasugrel (3.75 mg daily continued through outpatient period)

## Discussion

TCM is known to occur in a subset of patients with SAH. In some cases, it can lead to cardiogenic shock, complicating endovascular treatment and the management of cerebral vasospasm. The underlying mechanism is thought to involve excessive sympathetic stimulation, resulting in a catecholamine surge, leading to transient myocardial dysfunction without coronary artery lesions.

IABP stabilizes hemodynamics through balloon inflation and deflation synchronized with the cardiac cycle in the descending thoracic aorta, thereby increasing coronary perfusion, raising MAP, reducing left ventricular afterload and workload, decreasing myocardial oxygen consumption, and enhancing cardiac output [5]. IABP was selected over alternatives such as inotropes, Impella, or extracorporeal membrane oxygenation (ECMO). Use of Impella or ECMO would have required full ICU-level support and posed vascular access conflicts with neurointerventional procedures. In contrast, IABP allowed for hemodynamic stabilization with manageable invasiveness, making it more suitable within the available care environment. Following IABP initiation, the patient’s left ventricular ejection fraction improved from 25% to 50% by day 5, and to 70% by day 14 as confirmed by transthoracic echocardiography. Serum NT-proBNP levels decreased from 10,200 pg/mL at admission to 50 pg/mL by post-admission day 14. No new ST-T changes or elevation of cardiac enzymes were observed during the recovery phase. These parameters objectively support the reversal of cardiogenic shock and stabilization of cardiac function. However, when IABP is inserted via the right femoral artery, the commonly used ipsilateral femoral access for neuroendovascular procedures may be restricted, potentially influencing the choice of therapeutic strategy. While recent reports have demonstrated the feasibility of carotid artery stenting through the thoracic aorta at the site of IABP via transfemoral arterial access, there remains a significant risk of balloon rupture due to potential interference between the guiding catheter and the IABP balloon, necessitating careful procedural planning [6].

In the present case, surgical clipping was also considered; however, the need for anticoagulation during IABP support, the difficulty in interrupting the procedure should hemodynamic instability arise intraoperatively, and the possibility of accessing the aneurysm without interfering with the IABP via dTRA - all favored coil embolization. As a highly invasive procedure might have worsened cardiopulmonary function, this concern reinforced the decision to pursue endovascular coiling instead of surgical clipping.

Clazosentan, an endothelin receptor antagonist that inhibits cerebral vasospasm via the endothelin pathway, has demonstrated efficacy in reducing the incidence of cerebral vasospasm and related morbidity in a Phase III clinical trial and was approved for clinical use in Japan in 2022. However, it is associated with serious side effects such as fluid retention, pulmonary edema, and heart failure, which may necessitate treatment discontinuation. These adverse effects are attributed to its vasodilatory properties, which can induce hypotension and reduce cardiac output.

Nevertheless, given the importance of preventing cerebral vasospasm, several recent reports have highlighted the feasibility of continuing clazosentan therapy even in patients with cardiac dysfunction, provided that comprehensive systemic management is implemented. These studies emphasize that although drug-induced pulmonary edema is a significant concern, early intervention can mitigate this risk, and even after onset, careful use of dobutamine, strict fluid management, and optimized ventilatory settings may allow for continued therapy while maintaining circulatory and respiratory stability [7].

At our institution, where intensivists are not available, managing complex conditions resembling stroke-heart syndrome [8] poses certain limitations. Therefore, in anticipation of potential complications associated with clazosentan, we proactively initiated part or all of the so-called "Fantastic Four" heart failure medications - ARNIs, BBs, MRAs, and sodium-glucose cotransporter 2 inhibitors - before clazosentan administration. We have previously reported favorable outcomes with such a combination therapy. In this case as well, BBs (which are known to be effective for TCM), along with ARNIs, MRAs, and tolvaptan, were sequentially introduced, which may have contributed to the early improvement in cardiac function and a favorable clinical course [9]. While primarily an anti-epileptic agent, perampanel may have a theoretical benefit in preventing vasospasm via AMPA receptor antagonism, although the evidence remains limited [10]. Importantly, perampanel has not been shown to prevent angiographic vasospasm per se. However, a study by Suzuki et al. suggested that perampanel may reduce DCI and microinfarctions in SAH patients by mitigating neuroelectric disturbances affecting microcirculation [11]. 

Additionally, IABP support may enhance cerebral perfusion in patients with impaired cardiac function, potentially alleviating cerebral vasospasm [12]. In the present case, anticoagulation with argatroban alongside IABP support during the ultra-acute phase helped stabilize hemodynamics. The subsequent improvement in cardiac and respiratory function enabled a timely transition to clazosentan therapy. Unfractionated heparin was avoided due to concerns about hemorrhagic risk in the acute post-SAH period. Argatroban, a direct thrombin inhibitor, was selected for its better titrability and established safety profile in neurocritical care settings. Previous studies have suggested that argatroban may be a safe and effective alternative anticoagulant in critically ill patients requiring IABP support when heparin is contraindicated or preferably avoided [13]. In this case, APTT was strictly monitored and maintained at approximately 1.5 times the baseline value, ensuring bleeding risk was not exacerbated. This approach may have allowed for the simultaneous stabilization of systemic circulation, prevention of cerebral vasospasm, and suppression of delayed cerebral infarction.

Although the patient was managed outside the ICU, our institution operates a high-care unit (HCU) specifically designed for acute neurocritical cases. This unit is continuously staffed by neurosurgical physicians, with monitoring capabilities comparable to ICU standards. Continuous ECG, arterial pressure, and oxygen saturation monitoring were ensured, and daily cardiology consultations were incorporated to safely guide IABP and vasopressor management.

This report has inherent limitations, including its single-patient nature, lack of long-term follow-up, and absence of standardized vasospasm biomarkers. The extrapolation of treatment efficacy should therefore be approached with caution, and further multicenter studies are warranted to validate the safety and effectiveness of the integrated neuro-cardiac approach described herein.

## Conclusions

This case underscores the importance of individualized therapeutic strategies when managing aneurysmal SAH complicated by cardiogenic shock. The coordinated use of endovascular coiling, IABP, and tailored neurocardiac pharmacotherapy demonstrates how integrated, cross-disciplinary decision-making can optimize outcomes in complex, high-risk patients. Notably, the adoption of dTRA enabled safe neurointervention without interfering with femoral arterial IABP support, highlighting its value in critically ill patients requiring simultaneous cardiac and neurovascular management.
